# Management of Pediatric Post-renal Transplant Erythrocytosis With Enalapril: A Case Report

**DOI:** 10.7759/cureus.80850

**Published:** 2025-03-19

**Authors:** Msaada Nankumba, Beverly Schaefer, Mary O'Shea, Wayne R Waz, Xiaoyan Wu

**Affiliations:** 1 Internal Medicine-Pediatrics, University at Buffalo Jacobs School of Medicine and Biomedical Sciences, Buffalo, USA; 2 Pediatric Hematology, University at Buffalo Jacobs School of Medicine and Biomedical Sciences, Buffalo, USA; 3 Pediatric Nephrology, University at Buffalo Jacobs School of Medicine and Biomedical Sciences, Buffalo, USA

**Keywords:** angiotensin-converting enzyme inhibitors, erythrocytosis, pediatric kidney transplant, pediatrics hematology, post-transplant erythrocytosis

## Abstract

Post-transplant erythrocytosis (PTE) is a well-documented complication in adult renal transplant recipients but is less commonly reported in pediatric cases. We present an eight-year-old female patient with end-stage renal disease (ESRD) secondary to immune complex-mediated glomerulonephritis, who developed erythrocytosis 10 months after a deceased donor kidney transplant. Despite normal erythropoietin levels and mild obstructive sleep apnea, her hemoglobin (17 g/dL) and hematocrit (52%) remained elevated. She was initially treated with enalapril (2.5 mg daily), leading to hemoglobin normalization, later switched to losartan (titrated to 50 mg daily) for hypertension and proteinuria. Over five years, her hemoglobin has remained within the target range (11.5-14.5 g/dL), with controlled blood pressure and proteinuria. This case highlights the successful long-term management of pediatric PTE with renin-angiotensin system blockade while preserving graft function.

## Introduction

Post-transplant erythrocytosis (PTE) is a common hematological disorder observed in patients following renal transplantation. It is defined as a persistent elevation in hemoglobin (Hgb) (>17 g/dL) and hematocrit (Hct) (≥51%) levels, occurring mostly within the first two years after engraftment and lasting at least 3-6 months [1-3]. In adults, the incidence of PTE is around 10-22% in post-renal transplant patients [4,5] with some studies reporting it as high as 26% [6]. It is estimated that about 60% of patients with PTE experience symptoms such as malaise, headache, plethora, lethargy, and dizziness [4]. Vlahakos et al. report that the incidence of thromboembolic events in PTE patients ranges from 10% to 30% and about 2% mortality from associated complications [4].

The pathogenesis of PTE is multifactorial. While in some cases PTE can be attributed to excess erythropoietin (EPO) release from the native kidneys [7], there is evidence indicating that some PTE patients have similar or in some cases lower levels of EPO in comparison to control groups [8]. Additional factors implicated in its development include insulin-like growth factor-1 (IGF-1) and its binding proteins, activation of the angiotensin-II receptor, renal artery stenosis, and endogenous androgens. Therefore, at least three hormonal systems, EPO, the renin-angiotensin system (RAS), and endogenous androgens, play significant roles in PTE pathogenesis [4]. Ischemia in the native failed kidneys may stimulate RAS, leading to increased EPO secretion, which is not adequately suppressed by feedback mechanisms from elevated erythrocytosis [9]. Furthermore, androgens such as testosterone may amplify both angiotensin and EPO activities [3].

Risk factors for PTE include male gender, history of smoking, transplant renal artery stenosis, a rejection-free post-transplant course, and the presence of native kidneys [2,6]. Other studies including a meta-analysis indicated that significant additional risk factors for PTE include ganciclovir treatment after cytomegalovirus (CMV) infection, kidney from deceased donor, underlying polycystic kidney disease, and pre-transplant dialysis [10]. The use of cyclosporine immunosuppression was also identified as a significant risk factor for PTE in 1988 by the Tatman research team [11].

Management strategies for PTE described in the adult literature include phlebotomy and theophylline; however, these treatments carry risks such as iron deficiency anemia, infection, and other adverse effects. Angiotensin-converting enzyme inhibitors (ACEis), such as enalapril, and angiotensin receptor blockers (ARBs) have been shown to be safe and effective for the long-term treatment of PTE [12]. Nonetheless, the use and efficacy of ACEis in PTE have predominantly been reported in adults, with limited data available on their application in the pediatric population.

Here, we present a pediatric case of PTE.

This work was previously presented as a meeting abstract at the Eastern Society for Pediatric Research (ESPR) 33rd Annual Scientific Meeting in Philadelphia, Pennsylvania, on March 9-12, 2021 (Virtual).

## Case presentation

**Figure 1:**
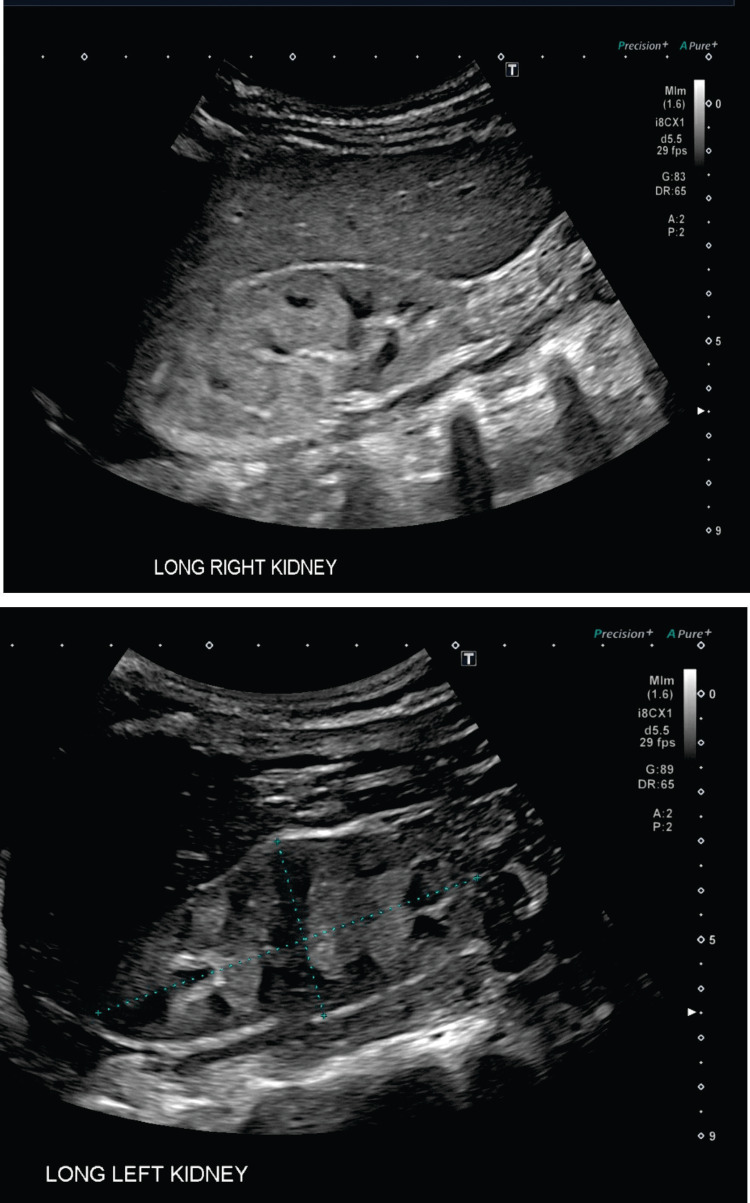
Renal ultrasound of native kidneys. The right kidney (upper) measures 7.7 × 3.0 × 4.3 cm. The left kidney (lower) measures 8.2 × 3.7 × 3.3 cm. The kidneys demonstrate increased parenchymal echogenicity with normal cortical thickness. There is no evidence of renal masses, calculi, or hydronephrosis.

A previously healthy six-year-old female patient was brought to the emergency department by emergency medical services (EMS) for altered mental status and gait disturbances. She had been in good health until two days prior when she developed upper respiratory symptoms, one episode of vomiting, and decreased urine output. On arrival, she was severely hypertensive (blood pressure (BP) 147/122 mmHg) and tachycardic (heart rate (HR) 124 bpm) and maintained normal oxygen saturation (SpO_2_ 98% in room air). Laboratory findings revealed severe azotemia, profound anemia, metabolic acidosis, and hyperkalemia (Table 1). A renal ultrasound revealed two native kidneys with increased parenchymal echogenicity without evidence of renal masses, calculi, or hydronephrosis (Figure 1). A kidney biopsy confirmed immune complex-mediated glomerulonephritis (data not shown). She was promptly initiated on hemodialysis.

**Table 1:** Laboratory findings at the diagnosis, on hemodialysis, and after receiving kidney transplant. BUN: blood urea nitrogen; sCr: serum creatinine; Hgb: hemoglobin; Tsat: transferrin saturation; ACEi: angiotensin-converting enzyme inhibitor

	At diagnosis (3/19/2018)	On hemodialysis (3/20/2018-6/3/2019)	Received kidney transplant (6/4/2019-6/2021)	
BUN (mg/dL)	>125	40-60	10-20	
sCr (mg/dL)	11.61	6-9	0.5-0.6	
Serum bicarbonate (mmol/l)	10	18-22	18-25	
Serum potassium (mmol/l)	7.4	3.5-5.5	3.5-5.5	
Serum phosphate (mg/dL)	9.7	3.5-5.5	3.5-5.5	
			Without ACEi (4/2020)	With ACEi (4/2020-4/2021)
Hgb (g/dL)	5.4	10.5-13.5	17	11.5-14.5
Ferritin (ng/mL) normal: 7-140 ng/mL	152	100-500	11	11-225
Iron (µg/dL) normal: 40-140 µg/dL	38	40-70	45	105
Tsat normal: 20-25%	14	15-30	11	11-32
Erythropoietin normal: 20-55 mU/mL	NA	NA	15.3	8.3-15.3

Although pediatric patients undergoing chronic hemodialysis face several potential complications, with cardiovascular collapse and infections being major causes of mortality, the patient in this case remained on chronic hemodialysis for 15 months without major complications (Table 1).

**Figure 2:**
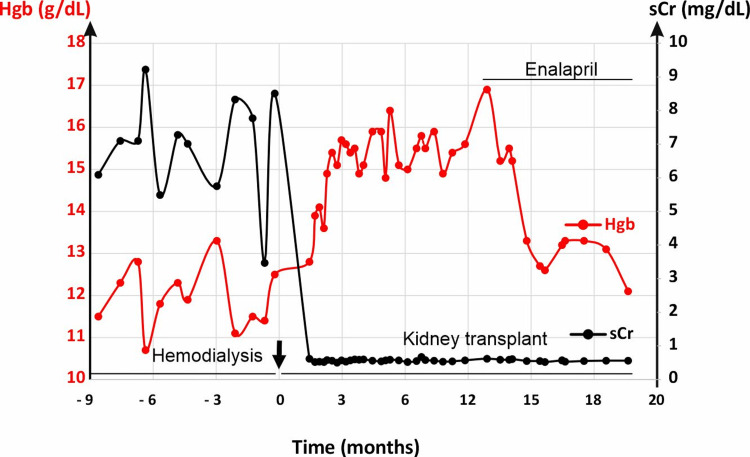
Effect of enalapril on post-transplant erythrocytosis. As indicated, during the chronic hemodialysis, sCr remained elevated, and Hgb was normal. After receiving a kidney transplant, sCr was normalized. Hgb was initially normal and started trending up after 10 months. Hgb was returned to normal with enalapril treatment with graft function being stable. Furthermore, the transition from enalapril to losartan did not affect Hgb levels (data not shown). sCr: serum creatinine; Hgb: hemoglobin

On June 4, 2019, at the age of eight years, she underwent a deceased donor kidney transplant (Figure 2). The transplant surgery was uncomplicated, and her native kidneys were retained. She received thymoglobulin and steroids for induction therapy and was started on mycophenolate and tacrolimus for immunosuppression. Postoperatively, her creatinine levels were within the normal range (0.5-0.6 mg/dL), and her estimated glomerular filtration rate (eGFR) ranged from 90 to 107 mL/min/1.73 m².

The patient experienced two episodes of CMV viremia (August-December 2019 and May-August 2020), for which she was treated with valganciclovir (Valcyte) 50 mg/mL, 9 mL twice daily. The CMV viremia resolved, and Valcyte was discontinued.

Ten months post-transplant, in April 2020, the patient was diagnosed with PTE, with a Hgb level of 17 g/dL, exceeding the normal range of 12.1-15.1 g/dL (Figure 2). Her serum creatinine (sCr), blood urea nitrogen (BUN), and electrolytes remained within normal limits (data not shown). At the time of PTE diagnosis, her serum levels of iron, ferritin, transferrin saturation, and EPO level were unremarkable (Table 1). A sleep study revealed episodes of oxygen desaturation, with a hypopnea index of 3.6 and a SpO_2_ of 92%.

The patient was started on enalapril 2.5 mg once daily, which was subsequently increased to 5 mg once daily for two years (Figure 2). She then changed enalapril to losartan 25 mg daily for BP control. Currently, she remains on losartan 50 mg daily. The treatment goal was to achieve a Hgb level of 11.5-14.5 g/dL and maintain a target BP below 111/73 mmHg and an optimal level of proteinuria.

Over the past five years, her Hgb stabilized at 11.5-14.5 g/dL, and her BP, urinary protein, and eGFR remained within the normal range. Aside from ACEis (enalapril) and ARBs (losartan), she continued immunosuppressive therapy to maintain graft function.

## Discussion

PTE is a well-documented complication following renal transplantation, primarily described in adult literature. PTE is defined as persistently elevated Hgb levels (>17 g/dL) and Hct levels (≥51%) after engraftment [1-3]. Several studies indicate that the incidence of PTE ranges from 8% to 26% of adult renal transplant recipients, typically developing 8-24 months post-transplant [4,5]. Approximately 1-2% of affected patients die from PTE-related complications [4]. The etiology of PTE is multifactorial, with at least four hormonal systems contributing to its pathogenesis: (1) EPO, (2) RAS, (3) testosterone, and (4) IGF-1 [3,4].

Management of PTE is crucial, as persistent PTE is associated with an increased risk of thrombotic events, as well as symptoms like malaise, headache, plethora, lethargy, and dizziness [13]. Regarding the frequency of specific symptoms in pediatric patients with PTE, data is limited. However, from an adult study conducted by Wickre et al., the incidence of thromboembolic events in PTE patients was 19%, i.e., there were 11 thromboembolic events reported to have occurred in 10 of the 53 patients with erythrocytosis in that study with none of such occurrences in the control group (P-value less than 0.001) [14]. Evidence suggests that maintaining Hct levels below 50-55% reduces these complications with the goal of treatment being to normalize Hgb levels in normotensive patients [15]. Traditional interventions, such as phlebotomy and theophylline, have limitations. Phlebotomy may lead to iron deficiency, while theophylline carries significant side effects.

ACEis, including enalapril, and ARBs are well-supported in the literature as safe and effective long-term treatments for PTE [12]. The mechanism by which ACEis reduce Hgb levels is thought to involve the accumulation of N-acetyl-seryl-aspartyl-lysyl-proline (Ac-SDKP), a tetrapeptide that inhibits erythropoiesis when ACE is inhibited [16,17]. Current Kidney Disease: Improving Global Outcomes (KDIGO) guidelines recommend using RAS blockers in kidney transplant recipients with proteinuria, hypertension, or erythrocytosis once stable graft function is achieved [18]. While ACEi use in PTE is well-documented in adult cases, data on its use in the pediatric population remain scarce.

In 1992, Krull et al. [19] reported a case of PTE in a seven-year-old child in Germany managed with a three-month course of theophylline, which failed to improve erythrocytosis, necessitating weekly phlebotomy to maintain Hct levels below 50%. This differs from our case, where enalapril effectively managed PTE without the need for phlebotomy. Weekly phlebotomy carries risks such as iron deficiency, patient discomfort, anxiety, challenges with vein access, and infection [20]. Compared to phlebotomy and theophylline, our report highlights that ACEis (enalapril) and ARBs (losartan) may offer a safe and effective therapy for pediatric PTE.

In 2015, Almonte et al. [2] reported a case similar to ours involving a 14-year-old girl in Mexico with a Hgb of 17.5 g/dL and Hct of 53% at 19 months post-transplant. She was treated with enalapril (0.13 mg/kg/day), which normalized her Hgb and Hct levels within six months. However, there are key differences between their case and ours. Almonte et al.'s patient received a kidney from a living donor, while ours received one from a deceased donor. Additionally, Almonte et al.'s patient took six months to achieve target Hgb levels, while our patient, treated with an initial enalapril dose of 2.5 mg/day (later increased to 5 mg/day), reached the target within two months. Notably, Almonte et al.'s patient experienced increased sCr and decreased eGFR, while our patient maintained stable renal function throughout treatment.

The paucity of data on ACEi use in pediatric PTE highlights the need for further research. Specifically, there is no standardized dose or duration of ACEi therapy for pediatric patients. This case underscores the potential of RAS inhibitors as effective treatments for pediatric PTE and calls for additional studies to establish evidence-based guidelines for their use.

## Conclusions

PTE is characterized by persistent Hgb levels above 17 g/dL and Hct levels exceeding 51%, typically developing within the first two years after engraftment and lasting at least 3-6 months. While more common in adults, PTE also occurs in pediatric transplant recipients and has clinical significance. Awareness and early intervention are crucial to preventing complications and preserving graft function.

Our case highlights the occurrence of PTE in a pediatric renal transplant patient and demonstrates successful long-term management using a renin-angiotensin-aldosterone system (RAAS) inhibitor. Enalapril effectively normalized Hgb levels without compromising graft function, which remained stable over five years of treatment. The patient continues on a maintenance dose of losartan (50 mg daily) with no recurrence of PTE.
